# Obesity and overweight: prevalence and associated socio demographic factors among mothers in three different areas in the Gaza Strip-Palestine: a cross-sectional study

**DOI:** 10.1186/2052-9538-1-7

**Published:** 2014-04-22

**Authors:** Rima Rafiq El Kishawi, Kah Leng Soo, Yehia Awad Abed, Wan Abdul Manan Wan Muda

**Affiliations:** School of Public Health, Al Quds University, Gaza, Gaza Strip, Palestine; Department of Nutrition, School of Health Sciences, Universiti Sains Malaysia, Health Campus, Kubang Kerian, Kelantan 16150 Malaysia

**Keywords:** Overweight, Obesity, Prevalence, Socio-demographic factors

## Abstract

**Background:**

The increasing number of obesity and overweight cases in developing countries, especially among women, requires serious attention because of its effects on the health care system and the quality of life. Few studies have been conducted in the Gaza Strip to determine obesity and overweight prevalence and the associated factors. This study aimed at determining the prevalence of obesity and overweight cases in relation to socio-demographic factors among mothers aged 18–50 years in the Gaza Strip-Palestine from June 2012 to September 2012.

Mothers childbearing age 18–50 years (n = 357) were selected using a cross-sectional multistage sampling methodology from three different geographical locations, namely, El Remal urban area, Jabalia refugee camp, and Al Qarrara rural area.

The weight and height of the mothers were measured, and their body mass indexes (BMI) were computed. The mothers were categorized according to the criteria of World Health Organization (WHO) for BMI. The criteria categorize mothers as overweight if they have a BMI 25–29.9 kg/m^2^ and obese if their BMI ≥ 30.0 kg/m^2^.

**Results:**

Obesity and overweight rates in urban area, refugee camp, and rural area were found to be 57.0%, 66.8%, and 67.5%, respectively. Moreover, BMI increased with age, adjusted b = 0.39; 95% CI (0.31, 0.48); *p* = < 0.001, whereas BMI was lower in low-income subjects, adjusted b = −1.59, 95% CI (−2.74,-0.44), *p* = 0.007. Housewives were more susceptible to obesity than employed woman, adjusted b = −2.76, 95% CI (−5.33,-0.19), *p* = 0.036. However, the study found no association among BMI level and household size, geographical location, educational level, and family assistance.

**Conclusions:**

The results showed that obesity and being overweight are highly prevalent among women in the Gaza Strip. Independent predictors of obesity in the population studied were increasing age, high income, and housewives. This finding is an important baseline for the monitoring of obesity and overweight cases in the future and highlights the need for community-based programs to combat this problem in Palestine.

## Background

The Palestinian National Authority consists of two geographically separated areas, namely, the Gaza Strip and West Bank. The total population of the Palestinian Territory was 2.72 million in the West Bank and 1.70 million in the Gaza Strip [1]. According to the Palestinian Central Bureau of Statistics, Palestinian refugees comprise 67.5% in the Gaza Strip [2]. In the past years, the rates of macro and micronutrient deficiencies increased as the Gaza Strip continued to suffer from closures, siege, destruction of infrastructure, and economic deterioration [3]. The pattern of non-communicable diseases (NCD_S_) has shifted tremendously in the most developing countries as compared to the industrialized regions of the world because of an increase in the rates of obesity and rapid emergence of metabolic syndrome. Obesity, which was detected among all socioeconomic and age groups in both developed and developing countries, may increase the risk of developing NCDs [4]. Obesity and being overweight constitute an important public health problem because they increase the risk of coronary heart disease, type 2 diabetes and certain types of cancers (e.g., breast, colon and endometrial) [5]. These diseases constitute as the major risk factor for morbidity and mortality in Palestine [6]. The pattern of dietary intake and the prevalence of obesity in developing countries, especially in low- and moderate-income countries, have changed immensely [7].

Middle Eastern countries have shown a later but faster-paced nutrition transition [7], and the Palestinian society has been undergoing nutrition shifts given its increased prevalence of obesity. A study of Palestinian adults estimated the prevalence of overweight and obesity as 58.7% and 71.3% among men and women respectively [8]. Many underlying etiological factors, namely, environmental, socio-economic, diet and physical activity transition play an important role in overweight and obesity prevalence and this has led to the deterioration of the socioeconomic status of the Palestinian [3]. Hence, this study aims to assess the prevalence of obesity and overweight cases among mothers, and the associated socio-demographic factors in three different geographical areas in the Gaza strip, namely, Jabalia refugee camp in the north, El Remal urban area in the middle, and Al Qarrara rural area in the south. To our knowledge, few studies on the prevalence of obesity among women in childbearing age in the Gaza Strip have already been conducted. However, this study addresses the patterns of obesity to obtain more information and serve as a guide for future planning of nutrition intervention programs that target this group and to help to curb this type of adult malnutrition. An earlier study reported that refugee camp and rural residency were higher in poverty and economic disparities than urban residency, we hypothesized that these factors are associated with a lower prevalence of overweight/obesity among mothers in rural area and refugee camp [9].

## Methods

### Study design, setting and population

This cross-sectional study assessed the weight status of mothers in the Gaza Strip by using anthropometric indices and survey results. This study was carried out for three months from June to September 2012.

### Sample size

The single proportion formula in the Epi-Info Software Revision (Version 2; 2002) was used to determine the required sample size and it came out to be 334. Anticipated population proportion (p) = 32.0%, which was for obesity prevalence among women in the Gaza Strip in 2010 [Al-Majdalawi J, Abed Y. Determinants of obesity among married women attended MCH clinics-Gaza Strip, unpublished Master thesis]. Expected population proportion was 37.0%, and level of significance = 5.0% (0.05). Accounting for 20.0% missing data, a total of 400 participants had to be recruited for the study. The researcher visited 357 out of 400 households in the three areas in the Gaza Strip, to obtain a response rate of 89.2%.

### Sampling method

Out of the total population in the three areas, females in the age group of 18–50 years comprised of 19.1% of the respondents. A total of 217, 100 and 40 females from Jabalia refugee camp, El Remal area-urban, and Al Qarrara rural area, respectively, were selected. A total of 357 households were identified and screened to find individuals who met the inclusion and exclusion criteria of being a mother, 18 to 50 years old and not-pregnant. Each of the districts in the Gaza City consisted of primary sampling units, clusters in each area containing 69,625 households in Gaza city, 6,167 households in Jabalia refugee camp, and 3,274 households in Al Qarrara [10]. The number of households chosen for each stratum was weighted in proportion to the total population of women aged 18–50 years in each urban, rural, and refugee camp area. At the first stage, a systematic sample of units was chosen from the urban area and refugee camp strata representing areas of high population density, then from the rural stratum, representing areas of lesser population density. At the second stage, random households were selected within each stratum in urban, rural and camp areas, respectively and mothers were then recruited from the selected households.

### Data collection

Consent from was obtained from each mother before interview. Moreover, the mothers were informed that the questionnaires could be answered voluntarily, anonymously and the information would be treated confidentially. The interviews took approximately 25 minutes. A structured questionnaire was used to collect socio-demographic information including location, age, level of education, household members, employment, monthly income, and household assistance.

The questionnaire was validated by taking expert opinion from seven professionals in the field of health and nutrition and necessary revisions were made based on their advices and comments. Prior to conducting the study, the questionnaire was pilot tested on 50 women and after obtaining the responses, necessary changes were made and the responses were evaluated.

Height was measured using a portable body meter with horizontal head-board attachment with 0.1 cm precision. The respondent stood against a wall without shoes, following the standard procedures of WHO [11]. Weight was determined by using calibrated portable digital weighing scale with 0.1 kg precision. Participants removed their shoes, socks, and all bulky clothing items. The BMI was computed with the following formula: weight (kg)/height (m)^2^. According to WHO, an adult BMI of less than 18.5 was considered to be under-weight, between 18.5 and 24.9 was normal, and between 25.0 and 29.9 was overweight. An adult with a BMI of 30.0 or higher was considered obese [12].

### Statistical analysis

We analysed our dataset with SPSS 20.0 (Statistical Package for the Social Sciences). We used means and percentages to describe the characteristics of the study sample. One-way analysis of variance (ANOVA) was performed to test for significant differences in mean of anthropometric measurements and age of mothers between the three different locations. Chi square test was performed to test for significant differences in socio-demographic characteristics in the three different locations. We used STAT software to perform Fisher exact test to examine the significant differences of BMI category in the three different locations. Linear regression was performed to control for confounding factors. In this model the dependent variable was BMI. We included the results of independent variables with *p* < 0.25, because they were considered important such as geographical place, age, level of education, household members, household assistance, monthly income, and employment. In the final model, the significance level was *p <* 0.05.

### Ethical issues

Necessary ethical clearance was obtained from the Ministry of Health and the Helsinki Committee in the Gaza Strip. Ethical clearance was also obtained from University Sains Malaysia (USM) ethical Committee.

## Results

A total of 400 households were selected and from these 357 mothers met the inclusion criteria. Among participants, 12 mothers refused to participate in the study, and we excluded 31 women who did not meet the inclusion criteria, 18 mothers were pregnant, and 13 households didn’t include mothers’ ages 18–50 years.

Socio-demographic characteristics and anthropometric characteristics of the participants are shown in Table 1. Mean age of mothers residing in urban area, rural area and refugee camp were 30.41, 28.73 and 31.36 years, respectively. The results revealed that three areas differed significantly in terms of age (*p* = 0.044). Similarly, significant difference was observed in terms of the number of household members present (*p* = 0.001). Most women in the three areas were unemployed (*p* = 0.56). The family monthly income was expressed in New Israeli Shekels (NIS); an NIS 3.9 was equal to US$1. Most households in the Gaza Strip were poor. There was no significant difference found in terms of monthly income (*p* = 0.425). More than half of households in Jabalia refugee camp received assistance including food or money, a significant difference in term of family assistance was found (*p* < 0.001). Moderate levels of education (i.e. preparatory and secondary) was the highest rate among participants in the three geographical locations compared to low level of education (i.e., elementary), and a high level of education (i.e., graduate and post-graduate) (*p* = 0.005).

**Table 1:** **The differences of anthropometric measurements and socio-demographic characteristics in the three different locations**

Variables	Urban n = 100		Rural n = 40		Refugee camp n = 217		P-value
	n (%)	Mean (SD)	n (%)	Mean (SD)	n (%)	Mean (SD)	
**Height (m)**		1.60 (0.06)		1.60 (0.06)		1.60 (0.05)	0.890^††^
**Weight (Kg)**		70.25 (18.20)		68.40 (12.95)		71.75(14.5)	0.390^††^
**Age (Year)**		30.40 (6.13)		28.73 (6.44)		31.36 (6.45)	0.044^††^
**Household members**		6.20 (1.90)		5.65 (2.10)		6.82 (1.97)	< 0.001^††^
**Working**							0.56^¶^
Housewife	93 (93.0)		38 (95.0)		208 (95.9)		
Employee	7 (7.0)		2 (5.0)		9 (9.1)		
**Monthly income (NIS)**							0.425^¶^
> 2000	37 (37.0)		9 (22.5)		63 (29.0)		
1000-2000	27 (27.0)		15 (37.5)		65 (30.0)		
< 1000	36 (36.0)		16 (40.0)		89 (41.0)		
**Household assistance**							< 0.001^¶^
Yes	23 (23.0)		14 (35.0)		148 (68.2)		
No	77 (77.0)		26 (65.0)		69 (31.8)		
**Education Level**							0.005^¶^
High	32 (32.0)		9 (22.5)		38 (17.5)		
Medium	67 (67.0)		26 (65.0)		16 (76.0)		
Low	1 (1.0)		5 (12.5)		14 (6.5)		

^††^One way ANOVA, ^¶^Pearson Chi-Square Test, Significant level at *p* < 0.05. (NIS) New Israeli Shekel, US$1 = 3.90 Shekel. Assistance: money or food.

In Table 2, most respondents were obese and overweight. The prevalence of obesity and being overweight was the highest in the rural area at 67.5%. The percentage of mothers who were obese or overweight in the refugee camp was almost the same at 66.8%. About 57.0% of women in the urban area were overweight or obese (*p* = 0.255).

**Table 2:** **Distribution of BMI category in the three different locations**

Variable	BMI	Normal weight	Underweight	Overweight	Obesity	Total	p-value
	Mean (SD)	(18.5-24.9)	(< 18.5)	(25.0-29.9)	(≥ 30.0)	n (%)	
City	27.44 (6.48)	41 (41.0)	2 (2.0)	26 (26.0)	31 (31.0)	100 (100.0)	0.255
Rural	26.94 (4.86)	12 (30.0)	1 (2.5)	19 (47.5)	8 (20.0)	40 (100.0)	
Refugee camp	28.28 (5.80)	68 (31.4)	4 (1.8)	78 (35.9)	67 (30.9)	217 (100.0)	

Fisher exact test, significant level at *p* < 0.05.

BMI classification [12].

In regard to age groups, obesity increased as age increased (*p* < 0.001), the highest prevalence of overweight in the age group (29–39) years, while in the age group (40–50) years obesity was showed a significantly highest prevalence (Table 3).

**Table 3:** **Distribution of BMI category among mothers’ age groups**

Variables	BMI	Normal weight (18.5-24.9)	Underweight (< 18.5)	Overweight (25.0-29.9)	Obesity (≥ 30.0)
Age group (year)	Mean (SD)	n = 121 (%)	n = 7 (%)	n = 123 (%)	n = 105 (%)
18-28	25.7 (5.0)	72 (49.0)	6 (4.1)	46 (31.3)	23 (15.6)
29-39	29.13 (5.79)	45 (26.0)	1 (0.6)	65 (37.6)	62 (35.8)
40-50	32.86 (6.2)	4 (10.8)	0 (0.0)	12 (32.4)	21 (56.8)

Pearson Chi-Square Test, Significant level at *p* < 0.05.

BMI classification [12].

In Table 4, Based on the univariate analysis, the factors that were significantly associated with BMI were household size (*p* = < 0.001), monthly income (*p* = 0.032), and age (*p* = < 0.001). We also included the results of independent variables with *p* < 0.25, because they were considered important and associated with factors. These variables included address, educational level, and household assistance. Moreover, we considered employment an important factor that may influence BMI. Results from multiple linear regression analysis showed a significant linear positive relationship was found between age and BMI. Mothers have a BMI that was 0.39 unit higher than those who were one year younger than them, adjusted b = 0.39, 95% CI (0.31, 0.48), *p* = < 0.001. A significant linear positive relationship between family monthly income and BMI. Mothers lived in households with a monthly income of less than 1,000 NIS have BMIs that were 1.59 units lower than those with high monthly income, which was more than 2,000 NIS, adjusted b = −1.59, 95% CI (−2.74,-0.44), *p* = 0.007. Finally, employed women were likely to have lower body weight than housewives, adjusted b = −2.76, 95% CI (−5.33,-0.19), *p* = 0.036. Addresses, the number of household members, and educational levels were not associated with BMI (all *p*-values > 0.05; results are not shown).

**Table 4:** **Socio demographic associated factors of BMI amongst women in the Gaza Strip**

Variables	Simple linear regression		Simple linear regression	
	b^a^(95% Cl)	P-value	b^b^(95% Cl)	P-value
Geographical location_0_	1.35 (−0.63,3.35)	0.186	-	-
Geographical location_1_	0.51 (−1.67,2.68)	0.647	-	-
Age (Year)	0.39 (0.30,0.47)	0.045	0.39 (0.31,0.48)	< 0.001
Educational level_0_	−0.57 (−2.05,0.91)	0.450	-	-
Educational level_1_	2.03 (−0.64,4.70)	0.140	-	-
Household members	1.05 (0.76,1.34)	<0.001	-	-
Household assistance	−0.92 (−2.15,0.31)	0.144	-	-
Monthly income_0_	0.91 (−0.44,2.27)	0.185	-	-
Monthly income_1_	−1.37 (−2.62,-0.12)	0.032	−1.59 (−2.74,-0.44)	0.007
Working				
Housewife (Reference)				
Employed mother	−1.24 (−4.06,1.56)	0.384	−2.76 (−5.33,-0.19)	0.036

^a^Crude regression coefficient, ^b^Adjusted regression coefficient.

Stepwise linear regression method applied. There were no interactions amongst independent variables.

Educational level_0_ is High level to medium and low level. Educationa_l_ level is Low level to medium and high level.

Income_0_ is > 2000 shekel to < 1000 and 1000-2000 shekel, Income_1_ is < 1000 shekel to > 2000 and 1000-2000 shekel.

Geographical location_0_ is urban to rural and refugee, Geographical location _1_ is Refugee to urban and rural area.

## Discussion

The prevalence of obese and overweight cases among women is high. Obesity is a serious public health problem in the Gaza Strip and must be emphasized because the trend is continuously increasing. Moreover, the prevalence of obesity and being overweight will contribute to the spread of NCD_S,_ such as diabetes, cardiovascular disease, and cancer, if no control measures are implemented. In the current study we hypothesized that the highest prevalence of overweight/obese women to be found in the urban area. Unexpectedly, the findings of this study reported that the highest prevalence of overweight/obese (BMI *≥* 25) women was found in rural area and the refugee camp; however, no significant association between obesity and being overweight with residency was found (*p* = 0.255). Our results indicated that approximately more than half of the women in the study area are overweight or obese. The rural area has the highest proportion of overweight and obese mothers 67.5%, followed by the refugee camp 66.8%, and urban area 57.0%. This could be attributed to higher consumption of less expensive and more energy-dense foods [13]. On other hand, a survey was conducted among women aged 40–65 years in Palestinian refugee camps in the West Bank, showed that high prevalence of obesity 70.0% [Rizkallah N. Coronary heartdisease risk factors among Palestinian women in refugee camps, unpublished PhD thesis]. Thus, these results might be explained by eating a greater of inadequate food especially who lived in the refugee camp, where food assistance programs provides food such as stocks of rice, flour, sugar, and cooking oil that is rich in sugar, carbohydrate, sodium, and fat which encourages weight gain [14]. According to the Nutritional Assessment of the West Bank and Gaza Strip in 2003, 71.8% of the population in the Gaza Strip especially in the refugee camp received food assistance from humanitarian agencies mainly from the United Nations Relief and Works Agency and the World Food Program [9]. Townsend et al., (2001) indicated that over consuming by food-insecure families when food assistance or money for food is available, followed by a short period of involuntary food restriction, followed by overeating, and could be a pattern that results in gradual weight gain over time [15]. This finding may predict a future increase in obesity trends unless appropriate preventive measures are carried out. Our findings were opposite to the results of other regional studies. The results in the Eastern Mediterranean Region (EMR) countries showed that prevalence of obesity and overweight is more prevalent in urban areas than in rural areas. The prevalence of obese and overweight women in urban area of Egypt was 84.8% as compared to 57.3% in rural areas. Previous study in Iran is also opposite to the findings of this study, that prevalence of overweight and obesity was higher in urban areas than rural areas [4].

On the other hand, our results showed that the prevalence of obesity (BMI *≥* 30) in the urban area was 31.0% higher than the rural area 20.0%. Similar to our results, previous Palestinian study reported that the BMI level among urban women was higher as compared to their rural counterparts. When adjusted by age, obesity prevalence (BMI *≥* 30) was 46.3% in urban and 35.0% in the rural area [16].

Regarding age groups, obesity increased as age increased our findings revealed that the highest prevalence of underweight in the first age group (18–28) years. Whereas, the highest prevalence of overweight/obesity was found among women in the period of middle of age, these results were similar to another local study [17].

Previous studies have indicated that the severity of obesity rise as age increases [18], and our results of a multiple linear regression showed that age was highly associated with obesity in women in all three areas, adjusted b = 0.39; 95% CI (0.31, 0.48); *p* = < 0.001. Obesity can occur at any age, but older women are more at risk. The association between obesity and age can be explained by the hormonal and postmenopausal changes experienced by women as well as their engagement in less active life styles [19].

Obesity is more prevalent among housewives than employed women, but no significant difference in obesity prevalence was found in the univariate analysis (*p* = 0.384). By contrast the multivariable analysis showed that housewives were at a higher risk for obesity with adjusted b = −2.7; 95% CI (−5.33, −0.19); *p* = < 0.036. This finding can be explained by the difference in physical activity levels, and food availability. These results are similar to the findings of previous studies [4, 20].

In the present study, no association between BMI and educational level was found. Some studies have argued that the risk of obesity and the level of education are inversely related particularly at high levels. A study in neighbouring Jordan found a negative association between educational level and obesity [21].

Our data showed that income significantly associated with BMI level, and the low rate of BMI level was seen in our participants with low monthly income, that is, earnings of less than 1000 NIS (*p* = 0.007). Generally, obesity has been associated with high socioeconomic status in middle and low income countries, including those in transition [22]. The results of the univariate analysis showed a significant association between BMI and household size (*p* = < 0.001). However, this association didn’t remain significant in the final model multivariable analysis, which showed no association between BMI level and household assistance (food or money).

## Conclusion

The results of this study showed that high prevalence of obesity and being overweight in the Gaza Strip can increase the likelihood for women to develop NCDs such as, type 2 diabetes mellitus, hypertension, heart disease and cancers (e.g., breast cancer) [5]. The increasing prevalence of obesity and overweight cases can be attributed to socio demographic factors such as age, high income, and housewives that lead to consume nutrients with high energy-dense food. This study is cross sectional and comprehensive, and thus, in-depth case control studies should be conducted in the future to address the risk factors of obesity in Palestine. Consequently, obesity must be reduced as well.
